# An experimental porcine model of invasive candidiasis

**DOI:** 10.1186/s40635-023-00514-6

**Published:** 2023-05-15

**Authors:** Anders Krifors, Anders Lignell, Miklós Lipcsey, Jan Sjölin, Markus Castegren

**Affiliations:** 1 Department of Physiology and Pharmacology, Karolinska Institutet, Stockholm, Sweden; 2 Centre for Clinical Research Västmanland, Uppsala University, Hospital of Västmanland, Västerås, Sweden; 3 Department of Medical Sciences, Section of Infectious Diseases, Uppsala University, Uppsala, Sweden; 4 Anaesthesiology and Intensive Care Medicine, Department of Surgical Sciences, Uppsala University, Uppsala, Sweden; 5 Hedenstierna Laboratory, Department of Surgical Sciences, Uppsala University, Uppsala, Sweden; 6 Centre for Clinical Research Sörmland, Uppsala University, Mälarsjukhuset, Eskilstuna Sweden; 7 Swedish Medical Products Agency, 751 03 Uppsala, Sweden

## Abstract

**Background:**

Invasive candidiasis (IC) is a severe and often fatal fungal infection that affects critically ill patients. The development of animal models that mimic human disease is essential for advancing our understanding of IC pathophysiology and testing experimental or novel treatments. We aimed to develop a large animal model of IC that could provide a much-needed addition to the widely used murine models.

**Results:**

A total of 25 pigs (including one control), aged between 9 and 12 weeks, with a median weight of 25.1 kg (IQR 24.1–26.2), were used to develop the porcine IC model. We present the setup, the results of the experiments, and the justification for the changes made to the model. The experiments were conducted in an intensive care setting, using clinically relevant anaesthesia, monitoring and interventions. The final model used corticosteroids, repeated *Candida* inoculation, and continuous endotoxin. The model consistently demonstrated quantifiable growth of *Candida* in blood and organs. The registered physiological data supported the development of the sepsis-induced circulatory distress observed in IC patients in the ICU.

**Conclusions:**

Our proposed porcine model of IC offers a potential new tool in the research of IC.

**Supplementary Information:**

The online version contains supplementary material available at 10.1186/s40635-023-00514-6.

## Background

Invasive candidiasis (IC) constitutes bloodstream infections with *Candida *spp. or deep-seated infections with or without concurrent candidemia [1]. *Candida *spp. are commensal pathogens to human microbiota but proliferate in the presence of immunosuppression, breach the skin or mucosal barrier, and cause invasive disease [2]. Patients in intensive care units account for most cases of IC, and significant risk factors include gastrointestinal surgery, central venous catheters, total parenteral nutrition and broad-spectrum antibiotic therapy [3]. IC is an increasingly identified nosocomial infection associated with significant mortality, morbidity, and costs [4]. Despite the availability of effective antifungal therapy, attributable mortality ranges from 20% to 40% [5, 6]. Clinical trials of IC are challenging to perform due to the relative scarcity of the infection and the heterogenous patient population at risk.

In vitro and in vivo experimental animal models of IC offer an alternative approach for evaluating mechanisms and potential interventions in IC. Standard animal models have substantially expanded our understanding of the pathophysiology of IC and can also be used to test antifungals or other adjunctive therapies. The in vivo models are also advantageous over less dynamic in vitro models by replicating complicated interactions between the pathogen and multiple physiological systems of the host. In contrast to clinical trials, identical strains of *Candida* can be used either from clinical isolates or modified strains, thus limiting the impact of strain-related virulence.

Murine models have extensively been used as effective and powerful tools in the study of IC. Several validated models are available and benefit from cost-effectiveness, ease of handling, and accessibility to genetically modified knockout mice [7–10]. While murine IC models offer several advantages, physiological and immunological differences to humans limit clinical translatability. For example, murine neutrophils are less effective in phagocytosing *C. albicans* than human neutrophils [11]. A large animal model of IC could provide a much-needed addition to the widely used murine models.

Pigs are frequently used due to their similarity with human anatomy, physiology, and size, which greatly benefit translational studies [12]. As an experimental model, pigs enable equipment and monitoring techniques relevant to intensive care. Only one porcine model of disseminated candidiasis has been published [13]. The model used gnotobiotic pigs orally inoculated with wild-type *C. albicans* and immunosuppressed through an intramuscular injection of methylprednisolone sodium succinate and oral cyclosporine. Disseminated candidiasis was confirmed by quantitative culturing of organ tissue samples. However, the model lacked blood culture data, a potential target for outcome measures. In addition, relevant data from respiratory and circulatory systems were not registered that could provide insights into the pathogenesis of IC.

In this study, we present the findings from a new experimental porcine model of IC. Our objective was to establish a model that consistently demonstrated blood culture and organ growth of *Candida* and displayed physiological changes consistent with IC. The experimental results were continuously evaluated to reduce the number of pigs needed to achieve this goal. The experiments were conducted in an intensive care setting, considering factors, such as anaesthesia, monitoring, and interventions. The proposed final model has the potential to serve as a foundation for further investigations into potential IC interventions.

## Methods

The experiment was approved by the Animal Ethics Board in Uppsala (Permit number: C230/11), and the animals were handled in accordance with the “Guide for the Care and Use of Laboratory Animals” (EU Directive 2010/63/EU). The experiments were conducted at the Hedenstierna Laboratory, Uppsala University, by experienced personnel in a setting resembling intensive care. The experiment, including all surgical procedures, was conducted under general anaesthesia, and all efforts were made to minimise animal suffering. The animals had access to food and water ad libitum until 1 h before the experiment. The study was designed with consideration of Minimum Quality Threshold in Pre-clinical Sepsis Studies (MQTiPSS) [14] and reported in adherence to the Animal Research: Reported of In Vivo Experiments (ARRIVE 2.0) guidelines [15].

### Anaesthesia and preparations

Before transportation and preparation, the pigs were sedated with an intramuscular injection of 50 mg xylazine (Bayer, Leverkusen, Germany). General anaesthesia was induced by an intramuscular injection of tiletamine–zolazepam (Virbac Laboratories, Carros, France) 6 mg × kg^−1^ and xylazine 2.2 mg × kg^−1^. A bolus dose of intravenously (i.v.) administered 20 mg morphine (Pfizer, Sollentuna, Sweden) and 100 mg ketamine (Pfizer) was given in conjunction with tracheal intubation. The animals were mechanically ventilated throughout the experiment. General anaesthesia was maintained by a continuous i.v. infusion of sodium pentobarbital (Apoteket, Umeå, Sweden) 8 mg × kg^−1^ × h^−1^, morphine 0.26 mg × kg^−1^ × h^−1^ dissolved in 2.5% glucose solution. Substitution of fluid losses was maintained by continuous i.v. administration of acetated Ringer's solution at a rate of 2 mL × kg^−1^ × h^−1^. All pigs received an initial perioperative dose of cefuroxime 750 mg i.v. (GlaxoSmithKline, Solna, Stockholm). The auricular peripheral and superior cava veins were catheterised using standard procedures. A cervical artery was catheterised under aseptic conditions using a 5 F catheter, and a central venous catheter was inserted in the external jugular vein using a 7 F Swan-Ganz catheter. The portal vein was catheterised by a 5 F catheter through splenic vessels accessible by a subcostal incision and evisceration of the spleen. Through a vesicostomy, a urine catheter was inserted into the urine bladder. After preparations were completed, the animals were stabilised for 30 min, during which a bolus of acetated Ringer's solution of 20 mL × kg^−1^ i.v. was administered. To decrease heat losses, a heating pad (Operatherm 200W, KanMed, Bromma, Sweden) was used and set to a temperature of 38 °C. The baseline was set to the end of stabilisation.

### Protocol

Initial mechanical ventilation settings included: respiratory rate 25 min^−1^, inspiratory–expiratory ratio 1:2, inspired oxygen fraction (FiO_2_) 0.3, positive end-expiratory pressure (PEEP) 5 cm H_2_O and tidal volume (*V*_*T*_) 10 mL × kg^−1^. *V*_*T*_ was adjusted before the start of the protocol to result in arterial partial pressure of carbon dioxide (PaCO_2_) of 5.0–5.5 kPa (38–41 mmHg). The ventilator was either a Servo 900C™ or a Servo I™ (Maquet, Stockholm, Sweden). Atelectasis formation was prevented by an alveolar recruitment manoeuvre and changing the animal's body position every third hour.

Heart rate (HR), mean arterial pressure (MAP), mean pulmonary arterial pressure (MPAP), and central venous pressure (CVP) were assessed continuously and registered hourly. In addition, cardiac output (CO)—measured by thermodilution, pulmonary capillary wedge pressure, core temperature, and urine production were recorded at the same intervals. Airway pressures and respiratory volumes were recorded hourly from ventilator readings. Arterial blood gases were obtained every 3 h and analysed for pH, gas tensions (PaO_2_, PaCO_2_), oxygen saturation, and haemoglobin (ABL 800 and Hemoximeter, Radiometer, Bronhoj, Denmark).

Vital parameters were maintained by interventions based on a protocol of preset limits (Table 1). The cardiac index was calculated by CO × (0.112 × weight^0.67^)^−1^, and the core temperature was maintained at 37.8–40.2 °C using blankets and heating pads.

**Table 1:** Intervention protocol

Parameter	Threshold values	Intervention
PaO_2_	< 10 kPa first time	Increase FiO_2_ to 0.6
PaO_2_	< 10 kPa after that	1. Increase FiO_2_ 0.6 → 0.8 → 1.0 AND 2. Increase PEEP 5 → 8 → 10 → 14 cm H_2_O AND 3. Lung recruitment manoeuvre
PaO_2_	> 30 kPa	Decrease FiO_2_ to next level 1.0 → 0.8 → 0.6 → 0.3
MAP and/or CI	MAP < 60 mm Hg and/or CI < 2.0 L × min^−2^ × m^−2^	Start norepinephrine infusion 0.07 µg × kg^−1^ × min^−1^. If ongoing norepinephrine infusion, increase the rate one step: 0.07 → 0.13 → 0.29 → 0.54 µg × kg^−1^ × min^−1^and measure cardiac output. If CO < 2.5 L x min^−1^, give a bolus dose of acetated Ringer's 15 mg × kg^−1^
MAP	MAP = MPAP (at < 90 min after baseline)	A single dose of 40 µg norepinephrine, i.v Repeat if needed
MAP	MAP = MPAP (at > 90 min after baseline)	1. A single dose of 20 µg norepinephrine, i.v AND 2. Start norepinephrine infusion 0.07 µg × kg^−1^ × min^−1^. If ongoing, increase rate one step: 0.07 → 0.13 → 0.29 → 0.54 µg × kg^−1^ × min^−1^ 3. A bolus dose of acetated Ringer's 15 mL × kg^−1^ at a rate of 750 mL × h^−1^
MAP	> 100 mm Hg	If ongoing norepinephrine infusion, decrease rate one step: 0.54 → 0.29 → 0.13 → 0.07 µg × kg^−1^ × min^−1^

### *Candida* species

The *C. albicans* strain SC5314 (ATCC catalogue no. MYA2876) was used in this study. The strain is a wild-type clinical isolate widely used in other animal models of invasive candidiasis (IC), and its genome has been fully sequenced. The inoculum was prepared by cultivating the *C. albicans* frozen stock on an SDA plate and incubating it at 35 °C for 2 days. The isolate was recultivated on an SDA plate and checked for homogenous growth and the absence of contamination. A single colony was then inoculated into 200 mL of yeast peptone dextrose (YPD). The day before the experiment, the inoculum was incubated at 35 °C in a shaker at 150 rpm for 18 h.

The inoculum was then centrifuged at 2500 rpm for 20 min. The supernatant was discarded, the inoculum was suspended in 20 mL of saline and was ready for administration. A dilution series of the inoculum was performed and cultivated for counting and ruling out bacterial contamination. The final concentration of *C.albicans* was around 108 CFU × mL^−1^. The inoculum was examined by microscopy and consistently showed buds, with no hyphae as signs of a logarithmic growth phase.

### Administration of the *Candida* inoculum

In all experiments, a *Candida* inoculum of 20 mL (approximately 2 × 10^9^ organisms) was administered one or multiple times through either a peripheral vein, a central venous catheter (CVC), intraarterially, or portal vein, using a syringe pump set to deliver the dose over 20 min.

### Prophylactic antibiotics

For experiments longer than 8 h, cefuroxime 750 mg was administered every eighth hour for the duration of the experiment.

### Endotoxin

Endotoxin, purified lipopolysaccharide (LPS) from *E. coli*: 0111:B4 (Sigma Chemical Co., St Louis, MO, USA) at a rate of approximately 0.07 μg × kg^−1^ × h^−1^ was administered to some animals to induce an inflammatory state resembling the clinical situation when invasive candidiasis occurs and to promote invasive disease [16].

### Corticosteroids

Methylprednisolone (Solu-Medrol: Pfizer, Sollentuna, Sweden) was administered at baseline to some animals at a 30 mg × kg^−1^ dose to induce immunosuppression and facilitate fungal growth.

### Blood and tissue cultures

Either of two methods was employed to obtain blood cultures:

### Pigs 1–18

A sample of 0.1 mL of arterial blood obtained under sterile conditions was smeared directly onto Sabouraud Dextrose Agar (SDA) plates and then incubated at 37 °C for 48 h. Fungal colonies were counted, and spot samples were smeared onto Mueller Hinton II agar for contamination testing.

### Pigs 19–24 and control

A sample of 10 mL of arterial blood was inserted into a blood culture bottle (BacT/ALERT FA FAN, bioMérieux, Stockholm, Sweden) and incubated using the BacT/ALERT system (bioMérieux, Stockholm, Sweden) for 96 h if the result was negative. The detection time for fungal growth was recorded. The blood culture bottles were spiked with vancomycin (Sandoz, Copenhagen, Denmark) to a concentration of 100 mg L^−1^ and gentamicin (Sandoz, Copenhagen, Denmark) to 5 mg L^−1^. Positive blood cultures were sub-cultured on SDA and Mueller Hinton II agar for bacterial contamination testing.

Blood cultures were obtained from all animals at baseline to exclude any preceding IC. At the end of the experiment, the animals were sacrificed through a combination of potassium chloride injection and the termination of mechanical ventilation. Post-mortem biopsies for fungal count were obtained from the lung, kidney, spleen, and liver under aseptic conditions. Tissue samples weighing approximately 1 g were homogenised with 3 mL of 0.9% saline using an Ultra Turrax high-speed homogeniser (Vevor, London, UK) for 15 s. A dilution series and sub-culturing were performed to quantify fungal growth.

### Statistics

Parameters are presented as mean and standard deviation or median and interquartile range. STATA version 16.1 (StataCorp, TX, USA) was used for the statistical analyses.

## Results

A total of 25 pigs (including one control) of both sexes, aged between 9 and 12 weeks, with a median weight of 25.1 kg (IQR 24.1–26.2), were used to develop the porcine IC model. We present the setup, the results of the experiments, and the justification for the changes made to the model.

### Pigs 1–4: intravenous/intra-arterial administration, 6 h experiment

**Fig. 1:**
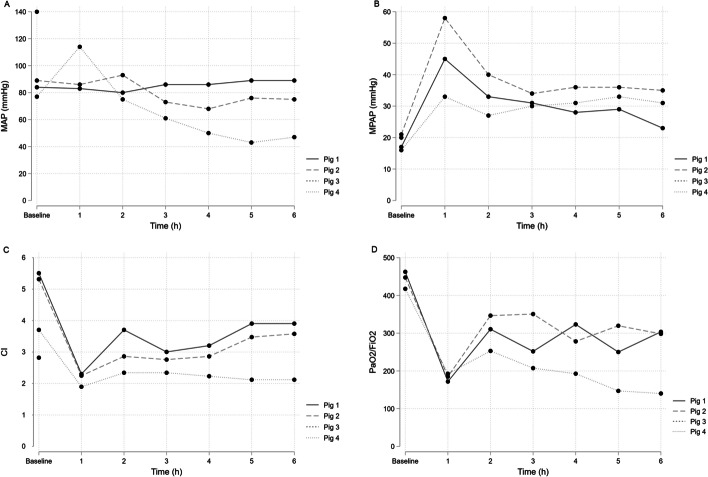
Pig 1–4 MAP, MPAP, CI and PaO_2_/FiO_2_. *MAP* mean arterial pressure, *MPAP* mean pulmonary arterial pressure, *CI* Cardiac Index

We arranged standard pig models and administered 20 mL (approximately 2 × 10^9^ organisms) at baseline through a central venous catheter (CVC) (Pig 1), peripheral vein (Pig 2, Pig 3), and intra-arterially (Pig 4). Blood cultures were obtained hourly throughout the experiment using agar plates, as described in the methods section. After the experiment, tissue samples from the lung, liver, spleen, and kidney were obtained and cultured according to the protocol (Additional file 1: Table S1). We observed a rapid rise in mean pulmonary artery pressure (MPAP), as presented in Fig. 1. The sudden increase in MPAP resulted in the cardiac arrest and death of Pig 3, and Pig 4 was hemodynamically unstable for the entire experiment. Growth of *Candida* was observed in all organs, particularly the lungs. Pig 4 had the highest fungal burden in the lungs compared to other tissues, despite the intra-arterial administration. Growth of *Candida* was observed in the blood of all animals 1 h from baseline. Only Pig 4 had *Candida* growth in blood in the later stages of the experiment.

### Pigs 5–8: portal vein administration, 6–30 h experiment

**Fig. 2:**
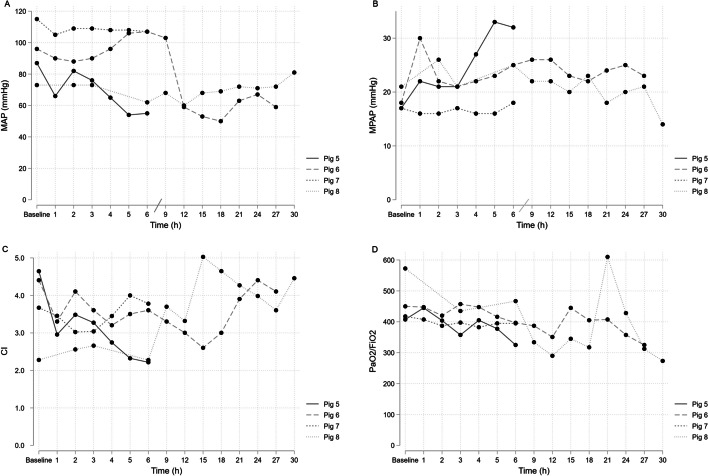
Pig 5–8 MAP, MPAP, CI and PaO_2_/FiO_2_. *MAP* mean arterial pressure, *MPAP* mean pulmonary arterial pressure, *CI* Cardiac Index

After a thorough analysis of the results of pigs 1–4, where a rapid increase in MPAP was observed following intra-venous/intra-arterial administration of *Candida* and resulted in severe circulatory instability, we decided to alter the route of infection to the portal vein. This method of infection mimics the clinical scenario of *Candida* translocation from the gastrointestinal tract. In contrast to the results obtained from pigs 1–4, a less pronounced increase in MPAP was noticed in pigs that received the inoculum through the portal vein (Fig. 2). To ensure that the conditions were favourable for the development of IC, we extended the duration of the experiment to 27 h and 30 h for Pig 6 and Pig 8, respectively. Despite these efforts, consistent blood culture growth using agar plates was not observed (Additional file 1: Table S1). However, Pig 8 exhibited massive growth of *Candida* in the liver at 30 h but no growth in other organs.

### Pigs 9–12: portal vein administration and endotoxin, 30 h experiment

**Fig. 3:**
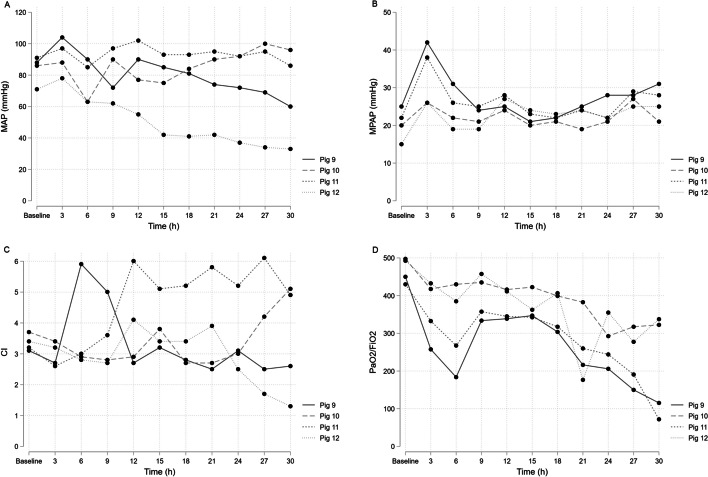
Pig 9–12 MAP, MPAP, CI and PaO_2_/FiO_2_. *MAP* mean arterial pressure, *MPAP* mean pulmonary arterial pressure, *CI* Cardiac Index

In an effort to achieve consistent growth of *Candida* in the blood, we added endotoxin (purified lipopolysaccharide (LPS) from *E. coli*) to the experiment. LPS regulates pro-, and anti-inflammatory cytokines and previous studies have shown that *E. coli* LPS can increase the virulence of *C. albicans* in experimental mouse models [17]. For Pigs 9 and 11, LPS was administered via continuous infusion at approximately 0.07 μg × kg^−1^ × h^−1^ throughout the experiment. For Pigs 10 and 12, the endotoxin infusion was discontinued after 6 h. While the addition of endotoxin led to a slight increase in positive blood cultures, consistent growth was still not achieved. However, all organs showed consistent growth, particularly the liver and lungs (Additional file 1: Table S1). Pig 12 experienced hemodynamic instability throughout the experiment and had positive results in half of the blood cultures, indicating IC as the primary cause (Fig. 3).

### Pigs 13–17: portal vein administration, corticosteroids and repeated *Candida* inoculation, 30–48 h experiment

**Fig. 4:**
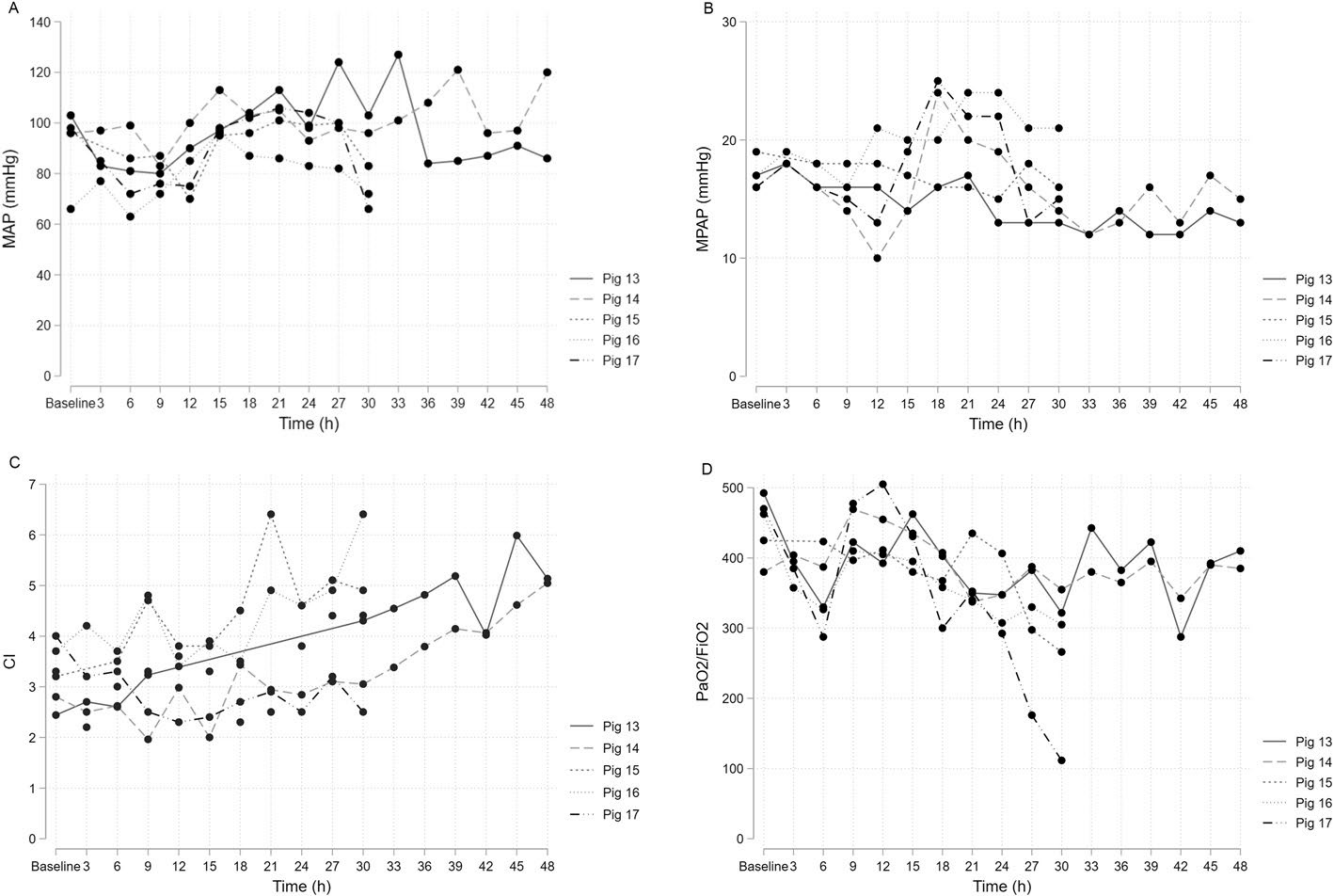
Pig 13–17 MAP, MPAP, CI and PaO_2_/FiO_2_. *MAP* mean arterial pressure, *MPAP* mean pulmonary arterial pressure, *CI* Cardiac Index

We hypothesised that increasing immunosuppression would result in more consistent growth in blood cultures; therefore, we added corticosteroids to the model. We administered 30 mg × kg^−1^ i.v. of methylprednisolone (Solu-Medrol, Pfizer, Sollentuna, Sweden) every 8 h to Pig 13 and at baseline to Pig 14, Pig 15, Pig 16 and Pig 17. To isolate the specific impact of corticosteroids, we opted not to use endotoxin in these animals. By excluding endotoxin from the experiments, we observed a decreased initial increase in MPAP compared to previous experiments. This could also be due to a balancing effect of the administered steroids on the observed rise in MPAP after *Candida* infusion. The addition of corticosteroids did not consistently lead to blood culture growth on agar plates. Furthermore, Pig 13 and Pig 14 remained hemodynamically stable throughout the prolonged 48-h experiment (Fig. 4). We hypothesised that repeated inoculation would increase the yield of *Candida* in blood cultures. In Pig 15, inoculation was repeated after 4 h, and we observed a general increase in positive blood cultures but no consistent growth (Additional file 1: Table S1). In Pig 16 and Pig 17, we repeated inoculation after nine and 24 h. Repeated inoculation resulted in an increase in fungal growth in all sampled organs but not consistent growth in blood (Additional file 1: Table S1).

### Final model Pigs 18–24 + control pig: repeated portal vein administration, corticosteroids, endotoxin, 30 h experiment

**Fig. 5:**
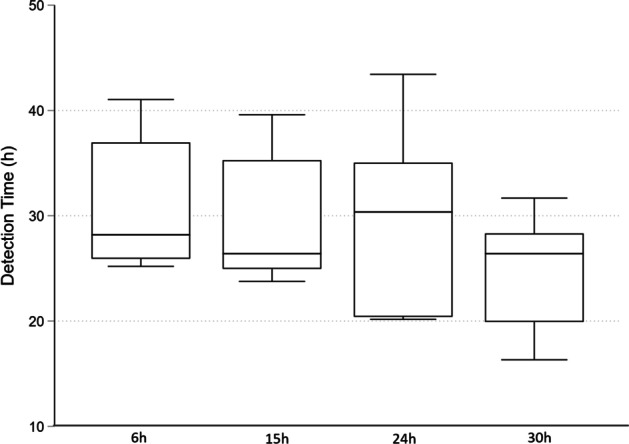
Blood culture detection time Pig 19–Pig 24. Boxplot—median, interquartile range and max–min

**Fig. 6:**
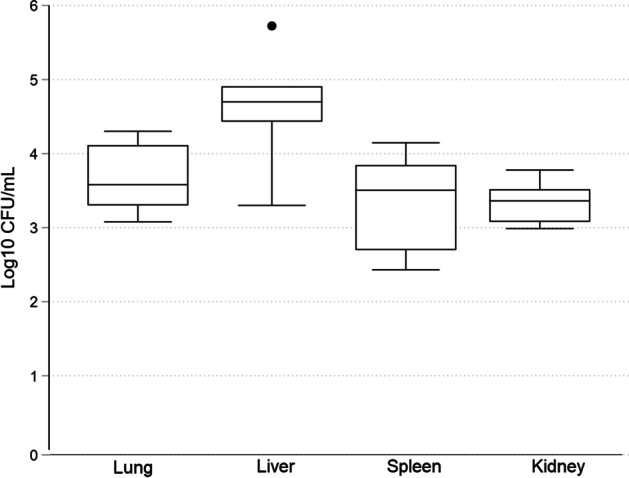
Organ growth of *Candida*. Boxplot—median, interquartile range and max–min, dot for outlier

The final model included a combination of endotoxin, corticosteroids, and repeated *Candida* inoculation (Table 2). For Pig 18, blood cultures were performed directly on agar plates, and for Pig 19–Pig 24 and the control, blood culture bottles were used as described in the methods section. Initially, some blood cultures were contaminated by bacteria (*Coagulase-negative Staphylococci* and *Enterococcus *spp.), making the detection time unreliable as bacteria generally grow faster and trigger the alarm. To prevent contamination, the blood culture bottles were spiked with vancomycin (Sandoz, Copenhagen, Denmark) to an internal concentration of 100 mg × L^−1^ and gentamicin (Sandoz, Copenhagen, Denmark) to an internal concentration of 5 mg × L^−1^. Efforts to lower the risk of contamination were also made, including changing CVC couplings, using waste tubes, and thorough disinfection. Still, two blood cultures (Pig 24, 24 h and 30 h) were contaminated, but the interventions significantly reduced the problem. We achieved consistent growth of *Candida* in blood cultures and all organs, where detection time and fungal burden in the organs could serve as outcome measures. The detection time for blood culture positivity was relatively stable but with high interindividual variability (Fig. 5). Physiological parameters and laboratory results for all pigs are presented as the means and standard deviations in Table 3. A boxplot of the organ growth is presented in Fig. 6. The average core temperature increased throughout the experiment. Thrombocytopenia and leukocytopenia developed from 6 to 30 h into the experiment. Circulatory effects were observed by an increased heart rate that continued for the entire experiment. Initially, there was a drop in MAP, but it recovered and was also observed in control. Static compliance in the respiratory system decreased over time, combined with reduced PaO_2_/FiO_2_. The control pig underwent the same procedures as the model animals, which included receiving corticosteroids at baseline and a continuous infusion of endotoxin. The catheter in the portal vein was used to administer 20 mL of saline solution instead of the *Candida* inoculum. The control pig maintained stable hemodynamics as indicated by constant HR, MAP, CI, and lactate levels. Respiratory parameters, including PaO_2_/FiO_2_ and static compliance, were also stable throughout the experiment.

**Table 2:** Final model: Pig 18–Pig 24 and control, characteristics and culture results

Animal	Sex (M/F)	Weight (kg)	Inoculum	LPS	BC CFU/ml ( ±)	Organ growth	(CFU/g)
Pig 18, 30 h	M	25.1	At baseline,9 h and 24 h, V. portae	∞	3 h: − 6 h: − 9 h: − 12 h: 30 15 h: 10 18 h: 10 21 h: − 24 h: − 27 h: 70 30 h: 90	Lung Liver Spleen Kidney	3.5 × 10^3^ 5.3 × 10^5^ 2.7 × 10^2^ 3.0 × 10^3^
Pig 19, 30 h	M	24.6	At baseline, 9 h and 24 h, V. portae	∞	3 h: (+) 6 h: (+) 9 h: + 12 h: (+) 15 h: (+) 18 h: + 21 h: + 24 h: + 27 h: + 30 h: +	Lung Liver Spleen Kidney	3.8 × 10^3^ 5.0 × 10^4^ 3.2 × 10^3^ 3.3 × 10^3^
Pig 20, 30 h	M	25.0	At baseline, 9 h and 24 h, V. portae	∞	3 h: + 6 h: (+) 9 h: + 12 h: + 15 h: (+) 18 h: + 21 h: + 24 h: + 27 h: + 30 h: (+)	Lung Liver Spleen Kidney	2.0 × 10^4^ 2.0 × 10^3^ 1.4 × 10^4^ 2.3 × 10^3^
Pig 21 30 h	M	24.4	At baseline, 9 h and 24 h V. portae	∞	6 h: + 15 h: + 24 h: + 30 h: +	Lung Liver Spleen Kidney	1.2 × 10^3^ 8.1 × 10^4^ 3.5 × 10^3^ 6.0 × 10^3^
Pig 22 30 h	M	23.4	At baseline, 9 h and 24 h V. portae	∞	6 h: + 15 h: + 24 h: (+) 30 h: ( +)	Lung Liver Spleen Kidney	1.3 × 10^4^ 4.0 × 10^4^ 7.0 × 10^3^ 1.2 × 10^3^
Pig 23 30 h	M	25.0	At baseline, 9 h and 24 h V. portae	∞	6 h: + 15 h: + 24 h: + 30 h: +	Lung Liver Spleen Kidney	2.0 × 10^3^ 6.8 × 10^4^ 8.6 × 10^2^ 1.4 × 10^3^
Pig 24 30 h	M	23.0	At baseline, 9 h and 24 h V. portae	∞	6 h: + 15 h: + 24 h: - 30 h: +	lung liver spleen kidney	8.5 × 10^3^ 2.7 × 10^4^ 5.0 × 10^2^ 9.7 × 10^2^
Control Pig 30 h	M	25.4	-	∞	6 h: - 15 h: - 24 h: - 30 h: -	lung liver spleen kidney	- - - -

*BC* blood culture, *CFU* colony forming units, *LPS* lipopolysaccharide, *∞* continuous infusion, + *Candida* growth, (+), bacterial contamination

**Table 3:** Physiological parameters and laboratory results for Pig 18–Pig 24 and control

	Baseline	3 h	6 h	9 h	12 h	15 h	18 h	21 h	24 h	27 h	30 h
HR ± SD (beats × min^−1^)	98 ± 33	100 ± 26	113 ± 36	103 ± 52	100 ± 36	98 ± 25	98 ± 25	106 ± 31	110 ± 21	128 ± 23	123 ± 31
Control	108	101	100	88	85	83	81	93	87	68	98
MAP ± SD (mmHg)	84 ± 14	87 ± 11	73 ± 14	82 ± 14	83 ± 11	76 ± 9	78 ± 11	85 ± 7	82 ± 12	85 ± 8	79 ± 12
Control	69	80	73	90	105	97	100	97	94	93	102
MPAP ± SD (mmHg)	21 ± 3	22 ± 4	20 ± 3	19 ± 3	20 ± 3	19 ± 3	19 ± 4	20 ± 7	22 ± 4	21 ± 7	23 ± 5
Control	21	23	21	20	20	20	18	18	16	18	18
CVP ± SD (mmHg)	8 ± 2	11 ± 6	11 ± 7	9 ± 1	9 ± 3	8 ± 4	8 ± 3	9 ± 4	9 ± 4	7 ± 4	9 ± 8
Control	6	6	9	8	11	10	10	7	7	7	7
Temp ± SD ©	38.6 ± 1.1	39.9 ± 2.3	40.8 ± 2.4	40.0 ± 2.1	39.6 ± 1.5	40.0 ± 1.4	40.4 ± 1.2	40.8 ± 0.8	41.3 ± 0.7	41.1 ± 0.6	40.6 ± 0.7
Control	38.1	39.4	40.1	39.9	40.2	40.1	40.1	40.2	40.7	40.5	40.3
CI ± SD	3.0 ± 0.8	3.1 ± 0.5	3.0 ± 0.4	3.5 ± 0.8	3.8 ± 1.6	3.8 ± 1.2	3.1 ± 1.6	3.8 ± 1.4	4.6 ± 1.7	4.3 ± 0.7	4.2 ± 0.6
Control	2.4	3.5	2.8	4.1	2.4	2.4	3.7	3.1	3.1	4.8	4.3
Compliance ± SD	33.6 ± 3.6	30.5 ± 4.5	27.8 ± 6.4	29.5 ± 5.5	27.8 ± 5.2	30.1 ± 4.8	26.2 ± 4.8	28.3 ± 5.8	24.1 ± 5.0	22.3 ± 6.7	20.0 ± 5.7
Control	31.6	27.5	27.6	31.0	31.3	27.5	27.4	35.8	27.1	27.3	26.9
PaO_2_/FiO_2_ ± SD	449 ± 44	435 ± 64	367 ± 80	419 ± 50	403 ± 35	414 ± 42	376 ± 36	386 ± 29	335 ± 58	389 ± 111	319 ± 91
Control	460	393	368	458	448	438	453	433	418	393	395
pH ± SD	7.47 ± 0.04	7.49 ± 0.04	7.48 ± 0.04	7.49 ± 0.03	7.47 ± 0.03	7.48 ± 0.04	7.48 ± 0.04	7.51 ± 0.05	7.50 ± 0.02	7.49 ± 0.04	7.49 ± 0.04
Control	7.46	7.47	7.47	7.51	7.50	7.50	7.53	7.50	7.53	7.51	7.52
Hb ± SD (g/L)	91 ± 8	96 ± 8	99 ± 28	89 ± 8	91 ± 8	83 ± 13	84 ± 10	84 ± 11	85 ± 16	85 ± 12	83 ± 9
Control	91	96	94	72	85	85	83	83	78	66	55
Glucose ± SD	9.2 ± 1.7	8.4 ± 1.0	7.9 ± 0.8	6.7 ± 2.4	6.7 ± 2.0	6.8 ± 1.6	7.2 ± 1.7	7.0 ± 2.2	6.4 ± 2.2	5.7 ± 1.9	5.2 ± 1.7
Control	10.6	8.5	8.9	8.3	7.6	7.5	7.1	6.2	5.9	5.5	5.5
urine (IQR) output/h		85 (70–200)	160 (110–1060	240 (50–440)	1060 (160–1200)	520 (420–625)	290 (200–760)	610 (300–740)	480 (410–900)	360 (180–770)	440 (200–515)
Control		1240	1180	200	340	280	340	400	1460	820	240
Lactate ± SD	2.0 ± 0.5	1.2 ± 0.2	1.1 ± 0.4	1.0 ± 0.3	1.2 ± 0.7	1.6 ± 1.3	1.4 ± 0.7	1.3 ± 0.3	1.2 ± 0.3	1.7 ± 1.2	1.7 ± 1.3
Control	2.1	0.9	1.0	0.7	0.8	0.8	0.6	0.6	0.6	0.7	0.8
Trc ± SD			381 ± 98								187 ± 96
Control			339								288
WBC ± SD			24.1 ± 7.8								6.8 ± 2.0
			19.6								4.9

*HR* heart rate, *SD* standard deviation, *MAP* mean arterial pressure, mean pulmonary arterial pressure, *CVP* central venous pressure, *CI* Cardiac Index, *Trc* trombocytes, *WBC* white blood cells, *CFU* colony forming units

## Discussion

This study presents the development process and results of a novel porcine model of IC. The model is a valuable contribution to the existing literature and an important addition to existing mice models. The proposed model demonstrated the most consistent results using a 30-h experiment with repeated portal vein inoculation of *Candida* in combination with immunosuppression with corticosteroids and continuous administration of endotoxin. Using this approach, we also achieved consistent and quantifiable growth of *Candida* in all sampled organs and the majority of blood cultures. The registered physiological data supported the development of the sepsis-induced circulatory and respiratory distress observed in IC patients in the ICU and was not observed in the control pig. Administration of *Candida* into the portal vein mimicked the translocation of *Candida* in the gastrointestinal tract to the circulation, a common route of infection in ICU patients.

Porcine models have become increasingly important in biomedical research due to their anatomical and physiological similarities to humans. They have been particularly useful in infectious disease research, as they are susceptible to many of the pathogens that also infect humans. Porcine models have been used to study viral infections, such as influenza, as well as bacterial infections, such as tuberculosis, pneumonia, wound infections, and sepsis [18–22]. Our model represents the first porcine IC model using monitoring and interventions relevant to intensive care, and could be an important first in the expanding use of porcine models.

Interestingly, after evaluating mouse models of IC, it was found that the fungal burden appears to decline in all organs over time except the kidney [23]. Lionakis et al. attributed this to the difference in leukocyte infiltration between organs [23]. They reported that *Candida* filamentation was observed in the kidneys but not the liver and spleen. Our study has shown consistent growth in all organs even after 48 h in Pig 13 and Pig 14. The formation of filaments is an attribute of *Candida spp.* with direct implications for tissue invasion and virulence and could be a potential explanation of the observed results, but data on filamentation was lacking in our study.

In Pigs 1–18, we cultured blood directly on agar plates, leading to mixed results that predominantly lacked consistent growth in blood. It has been reported that candidemia commonly has a fungal burden of < 1 CFU × mL^−1^. Therefore, the sensitivity of using only 0.1 mL of blood is severely hampered. We initially employed this approach due to the lack of access to a bottle culturing system. When we shifted our approach to blood culture bottles, we achieved substantially higher yields, initially at the expense of bacterial contamination. We made efforts to decrease the risk of bacterial contamination (change of CVC couplings, use of waste tubes, and thorough disinfection). However, this approach was also insufficient, and we prevented this problem by adding antibiotics to the blood culture bottles. A disadvantage of using blood culture bottles is that the fungal burden cannot be quantified, but the detection time could be used as a surrogate marker [24].

The use of endotoxin in our model may be debatable and is not recommended by the MQTIPSS consensus initiative [14]. The effects of endotoxin on the immune system vary depending on the dose and timing of administration. Endotoxin-tolerant macrophages shift from pro-inflammatory to anti-inflammatory phenotypes over time, characterized by a reduction in pro-inflammatory cytokines such as tumor necrosis factor alpha (TNFα), interleukin (IL)-12, and IL-6, and an increase in anti-inflammatory cytokines such as IL-10 and transforming growth factor beta (TGFβ) [25]. Grondman et al. [26] found that monocytes in healthy volunteers had a reduced *Candida* killing capacity after initial exposure to endotoxin. In our final model, we utilized a continuous infusion of endotoxin to resemble the anti-inflammatory state in the later stages of sepsis when the risk of secondary fungal infection is highest.

Throughout the experiments, we observed some interesting findings. The administration of *Candida* inoculum directly into a central vein or artery resulted in a rapid increase in MPAP and subsequent circulatory collapse. The effect was sudden, making micro-clotting of the lung capillaries less likely, and a receptor effect triggering vasoconstriction a more probable explanation. Endothelin, a potent vasoconstrictor produced in the porcine lung endothelium, could be the culprit, as the release of endothelin mediates strong capillary smooth muscle activation in porcine lungs [27]. An embolic event is also plausible, but further investigation is needed to discover the underlying mechanism.

There are many obstacles to performing prospective randomised trials in critically ill patients. Therefore, clinically relevant animal models could be of additional value. Our proposed model could be used for translational studies in the context of host interactions and interventions before human studies.

### Limitations

There are several limitations to our IC model. Significant inter-individual variations in physiological measures and microbiological results were evident by large standard deviations. Although not necessarily a problem and shared in biological studies, interventional studies would require larger groups of animals, which may not always be feasible due to extensive workload and costs.

## Conclusions

In conclusion, our porcine model of IC offers a potential new tool in the research of IC and aspires to advance our understanding of IC pathophysiology, support future trial designs, and test experimental or novel treatments and interventions.

## Supplementary Information

Additional file 1: Table S1. Physiological parameters and laboratory results for Pig 11–Pig 17.

## Data Availability

The data sets used are available from the corresponding author on reasonable request.

## Abbreviations

CFU: Colony forming units
CI: Cardiac Index
CS: Corticosteroids
CVC: Central venous catheter
CVP: Central venous pressure
HR: Heart rate
IC: Invasive candidiasis
ICU: Intensive care unit
LPS: Lipopolysaccharide
MAP: Mean arterial pressure
MPAP: Mean pulmonary arterial pressure
PEEP: Positive end-expiratory pressure
SDA: Sabourad dextrose agar
Trc: Thrombocytes
WBC: White blood cells
