# Supplementary material for: An experimental porcine model of invasive candidiasis
Source: Intensive Care Med Exp. 2023 May 15;11:27. doi: 10.1186/s40635-023-00514-6 (PMC10183382; doi:10.1186/s40635-023-00514-6)
Supplement: Supplementary file 1 — Additional file 1: Table S1. Physiological parameters and laboratory results for Pig 11–Pig 17. [file 40635_2023_514_MOESM1_ESM.docx]

Supplementary Table 1. Physiological parameters and laboratory results for Pig 11 – Pig 17

| Animal | Sex (M/F) | Weight (kg) | Inoculum | LPS | CS | BC (CFU/mL) | Organ growth |  |
| --- | --- | --- | --- | --- | --- | --- | --- | --- |
| Pig 1,   6 h | M | 26.2 | At baseline, CVC | - | - | 0 h -  1 h >500   2 h -  3 h -  4 h -  5 h -  6 h - | lung  liver  spleen  kidney | +++  +  +  + |
| Pig 2,   6 h | M | 25.4 | At baseline, peripheral vein | . | - | 0 h -  1 h >500   2 h -  3 h -  4 h -  5 h -  6 h - | lung  liver  spleen  kidney | +++  +  +  + |
| Pig 3,   6 h | M | 21.9 | At baseline, peripheral vein | - | - | 0 h died | lung  liver  spleen  kidney | na  na  na  na |
| Pig 4,   6 h | M | 22.3 | At baseline, intraarterial | - | - | 0 h -  1 h >500   2 h 100   3 h 50 4 h 40   5 h -  6 h 60 | lung  liver  spleen  kidney | +++++  +++  ++  ++ |
| Pig 5,   6 h | M | 24.2 | At baseline, v. porta | - | - | 0 h -  1 h >500   2 h -  3 h -  4 h -  5 h -  6 h 10 | lung  liver  spleen  kidney | +++  +  +  + |
| Pig 6, 27 h | M | 26.2 | At baseline, v. porta | - | - | 0 h -  1 h -  2 h -  3 h -  4 h -  5 h -  6 h -  9 h -  12 h -  15 h -  18 h -   21 h -  24 h -  27 h - | lung  liver  spleen  kidney | na  na  na  na |
| Pig 7,  6 h | M | 23.4 | At baseline, v. porta | - | - | 0 h-  1 h -  2 h -  3 h -  4 h -  5 h -  6 h - | lung  liver  spleen  kidney | -  +++  -  ++ |
| Pig 8, 30 h | F | 28.4 | At baseline, v.porta | - | - | 0 h  1 h -  2 h -  3 h 190  4 h -  5 h -  6 h -  9 h -  12 h -  15 h -  18 h -   21 h -  24 h -  27 h -  30 h - | lung  liver  spleen  kidney | -  ++++  -  - |
| Pig 9, 30 h | M | 26.1 | At baseline, v.porta | ∞ | - | 0 h -  3 h 190  6 h -  9 h -  12 h -  15 h -  18 h -   21 h -  24 h -  27 h -  30 h - | lung  liver  spleen  kidney | +  ++++  +  + |
| Pig 10, 30 h | F | 26.0 | At baseline, v.porta | 6h | - | 0 h -  3 h -  6 h 200  9 h -  12 h -  15 h -  18 h -   21 h -  24 h -  27 h >500  30 h - | lung  liver  spleen  kidney | +++  ++++  +  + |
| Pig 11,  30 h | M | 29.0 | At baseline, v. porta | ∞ | - | 0 h -  3 h -  6 h -  9 h -  12 h -  15 h 10  18 h -   21 h -  24 h -  27 h 20  30 h - | lung  liver  spleen  kidney | +++  ++++  +  + |
| Pig 12, 30 h | M | 25.7 | At baseline, v.porta | 6h | - | 0 h -  3 h -  6 h 10  9 h -  12 h -   15 h 80  18 h 110  21 h 60  24 h 10  27 h -  30 h - | lung  liver  spleen  kidney | ++  ++++  +  + |
| Pig 13, 48 h | M | 24.6 | At baseline, v.porta | - | 30 mg/kg t.i.d. | 3 h: na  6 h: >500  9 h: >500  12 h: 80  15 h: 120  18 h: 90  21 h: -  24 h: 20  27 h: 10  30 h: 250  33 h: 50  36 h: -  39 h: -  42 h: -  45 h: -  48 h: 20 | lung  liver  spleen  kidney | ++  ++++  ++  ++ |
| Pig 14 48 h | M | 27.0 | At baseline, v.porta | - | 30 mg/kg at baseline | 3 h: 20  6 h: na  9 h: -  12 h: -  15 h:>500  18 h: 10  21 h: -  24 h: 10  27 h: -  30 h: -  33 h: 20  36 h: -  39 h: -  42 h: -  45 h: -  48 h: - | lung  liver  spleen  kidney | ++  ++++  ++  ++ |
| Pig 15, 30 h | M | 27.0 | At baseline and 4 h, v.porta | - | 30 mg/kg at baseline. | 0 h: -  3 h: -  6 h: >500  9 h: 400  12 h: >500  15 h: -  18 h: -  21 h: >500 24 h: -  27 h: -  30 h: - | lung  liver  spleen  kidney | ++++  ++++  ++  ++ |
| Pig 16, 30 h | M | 23.9 | At baseline, 9h and 24h, v.porta | - | 30 mg/kg at baseline | 3 h: 100  6 h: -  9 h: -  12 h: >500  15 h: -  18 h: 20  21 h: -  24 h: -  27 h: -  30 h: 50 | lung  liver  spleen  kidney | ++++  ++++  ++++  ++++ |
| Pig 17 30 h | M | 27.0 | At baseline, 9h and 24h, v.porta | - | 30 mg/kg at baseline | 3h: -  6h: 10  9h: 90  12 h: -  15 h: -  18 h: -  21 h: -  24 h: -  27 h: -  30 h: - | lung  liver  spleen  kidney | ++++  ++++  ++++  ++++ ­­­ |
| LPS – Lipopolysaccharide, CS – Corticosteriods (methylprednisolone), CFU – Colony Forming Units, ∞ - continuous infusion, t.i.d. – three times daily, + (1-50 CFU/g), ++ (51-250 CFU/g), +++ (251-500 CFU/g), ++++ (>500 CFU/g) | | | | | | | | |
